# [^11^C]Fentanyl: radiosynthesis and preclinical pet imaging for its pharmacokinetics

**DOI:** 10.1186/s41181-025-00394-z

**Published:** 2025-10-28

**Authors:** Woochan Kim, Aaron K. Wozniak, Nathaniel J. Burkard, Michael L. Freaney, Ailen Costamagna-Soto, Kelly A. O’Conor, Abolghasem Bakhoda, Seth M. Eisenberg, Wenjing Zhao, Jeih-San Liow, Nora D. Volkow, Sung Won Kim

**Affiliations:** 1 Laboratory of Neuroimaging, National Institute On Alcohol Abuse and Alcoholism, National Institutes of Health, Bethesda, MD 20892 USA; 2 Molecular Imaging Branch, National Institute of Mental Health, National Institutes of Health, Bethesda, MD 20892 USA

**Keywords:** Fentanyl, Carbon-11, Positron emission tomography, Pharmacokinetics

## Abstract

**Background:**

Fentanyl is a potent synthetic opioid widely used for pain management and anesthesia, but the high prevalence of its misuse and its key contribution to overdose fatalities in the United States have made it a major drug of concern. Although fentanyl’s onset, duration, and toxicity depend on its pharmacokinetics and specific tissue distribution, most studies have focused primarily on plasma concentrations, leaving its distribution in critical tissues largely unexplored (this knowledge gap limits our understanding of fentanyl’s clinical effects, tissue accumulation, and the factors influencing its efficacy and safety). Here, we report the radiosynthesis of [^11^C]fentanyl for PET imaging and present a preliminary whole-body pharmacokinetic study in rodents.

**Results:**

[^11^C]Fentanyl was synthesized in 42 min in a high radiochemical yield (10.4 ± 5.7%, n = 5), radiochemical purity (> 99%), and molar activity (up to 2571.5 GBq/µmol at EOB). *N*,*N*-Diisopropylethylamine in chloroform was optimal for amidation. PET imaging in rats revealed rapid brain uptake (SUV_max_ 2.71 ± 1.04 g/mL) and fast washout (T_1/2_ = 5.06 min), both significantly increased by efflux transporter inhibition or knockout. Peripherally, high and prolonged uptake in adipose tissues was observed (SUV_max_ = 1.73 ± 0.313 g/mL, T_1/2_ = 177 min), with > 60% of C-11 remaining as unchanged [^11^C]fentanyl at 60 min.

**Conclusions:**

We successfully developed and automated the radiosynthesis of [^11^C]fentanyl, enabling PET imaging that revealed rapid brain kinetics and a critical role of P-gp/BCRP efflux in fentanyl disposition in brain. Prolonged retention in adipose tissue may delay brain clearance, potentially increasing the risk of re-narcotization (as has been reported in clinical cases after naloxone reversal). These findings advance our ability to quantify fentanyl tissue distribution and pharmacokinetics in the brain and body and provide a valuable tool for further studies in preclinical and clinical settings.

**Supplementary Information:**

The online version contains supplementary material available at 10.1186/s41181-025-00394-z.

## Introduction

Fentanyl is a potent synthetic mu-opioid receptor (MOR) agonist, widely used in clinical settings not only as an adjunct in anesthesia but also for managing acute post-operative pain and breakthrough cancer pain (Stanley 2005). The rise of illicitly manufactured fentanyl has led to widespread misuse, making it the main driver of the devastating overdose crisis in the United States. In 2022, fentanyl and analogues were involved in nearly 74,000 drug overdose deaths (CDC overdose prevention. About overdose prevention. 2025). The severity of fentanyl-related overdoses has been exacerbated by its mixture with other drugs such as heroin, cocaine, and methamphetamine, often consumed unknowingly by users, which has significantly complicated overdose reversal efforts (Volkow et al. 2023).

Given fentanyl’s critical role both in medicine and in the overdose crisis, investigating its pharmacokinetics has been essential for optimizing its therapeutic use (Peng and Sandler 1999) while also enhancing our understanding of overdose mechanisms, ultimately informing more effective management strategies (Baird et al. 2024; Chaudun et al. 2024). For instance, preclinical and clinical investigations of fentanyl’s pharmacokinetics have helped to optimize dosages for various patient populations (Okada et al. 2024; Shibutani et al. 2005). However, these studies mostly concern fentanyl’s dosages relevant to anesthesia and analgesia rather than patterns reported and observed in individuals who misuse fentanyl outside of medical settings. Another important consideration is the observation among some misusers of a secondary fentanyl peak, with an abrupt increase in fentanyl plasma concentration and associated respiratory depression. A previous study found that over a 240-min period, healthy volunteers injected with 0.5 mg fentanyl IV showed secondary peaks in plasma concentration between 45 and 90 min after administration (Stoeckel et al. 1982). Fentanyl’s pharmacokinetics are also impacted by demographic and clinical characteristics of a user, including age, obesity, metabolic function, among others. These factors, in addition to an individual’s history of fentanyl misuse, may drastically alter its pharmacokinetics and consequently, its physiological effects and overdose risk (Bird et al. 2023).

Preclinical studies using animal models have been crucial in exploring the threshold doses for severe respiratory depression associated with fentanyl overdose and addiction. These investigations provide valuable insights into the physiological effects of fentanyl at different plasma concentrations and can inform strategies for intervention. However, translating these findings to human physiology would benefit from in vivo non-invasive methods for direct measurement of fentanyl’s biodistribution and kinetics in the brain and body.

Positron emission tomography (PET) is a powerful quantitative imaging technique that allows for the in vivo measurement of drug concentrations and distributions in target tissues using radiolabeled compounds. Unlike conventional pharmacokinetic studies that rely on plasma drug concentrations, PET provides direct visualization and quantification of drug levels in tissues relevant to both therapeutic effects and adverse events, such as the brain (Ghosh et al. 2022). This non-invasive approach offers significant advantages for translational research, enabling repeated measurements and flexible experimental designs in laboratory animals and in humans, which would be particularly valuable for understanding the rapid onset and duration of fentanyl’s effects.

While early studies utilized H-3 and C-14 labeled fentanyl to investigate its metabolism and biodistribution in preclinical models, these radiotracers are not optimal as each subject has to be scarified for each time point (Schneider and Brune 1985; Nami et al. 2018). Therefore, a critical gap exists in our ability to non-invasively quantify fentanyl concentrations and kinetics in brain and other organs with PET that could also be eventually used for studies in humans. To address this limitation, we herein report the radiosynthesis of carbon-11 labeled fentanyl and present preliminary PET studies conducted in rodents, paving the way for translational pharmacokinetic investigations in humans.

## Materials and methods

### Materials

4-Anilino-*N*-phenethyl-piperidine (4-ANPP) was purchased from Cayman Chemical. The aqueous hydrochloric acid solution (2 N, RICCA Chemical Company, TX) was diluted with water for semi-preparative HPLC. Absolute ethanol and sodium phosphate buffer (45 mM phosphate, 60 mEq sodium) were obtained from Warner-Graham Company and Hospira Inc., respectively. Tetrahydrofuran (THF) was purified by distillation with sodium (dispersion in mineral oil, Strem Chemicals). All the other chemicals were purchased from Sigma-Aldrich (St. Louis, MO) and were used without any further purification.

Radiosynthesis was fully carried out and optimized with a commercially available module (Synthra MeIPlus Research). Radiochemical purity and molar activity were determined using an Agilent 1100 Series HPLC system (column, Agilent Eclipse XDB C-18 column, 150 × 4.6 mm, 5 µm; mobile phase, isocratic 0.1% trifluoroacetic acid solution/acetonitrile = 70/30; flow rate, 1 mL/min; detection wavelength, 210 nm) and a radiometric detector equipped with a B-FC-4100 BGO High Voltage Detector.

Animal use and protocol were approved by the Institutional Animal Care & Use Committee (National Institutes of Mental Health; MIB-03, MIB-04). Wistar rats (male; 297 ± 39.9 g, Envigo, Indianapolis, IN) were used for PET studies. P-gp and BCRP KO mice (female, 25.7 ± 2.22 g, bred in-house) and FVB mice (female, 25.3 ± 0.985 g, Charles River Laboratories, Wilmington, MA) were used to examine P-gp and BCRP influence on fentanyl pharmacokinetics. Both rats and mice were housed under a 12-h light/dark cycle. An LFER 150 PET/CT Scanner (Mediso Ltd., Budapest, Hungary) was used for dynamic PET study.

### Radiosynthesis of [^11^C]fentanyl

**Fig. 1:**
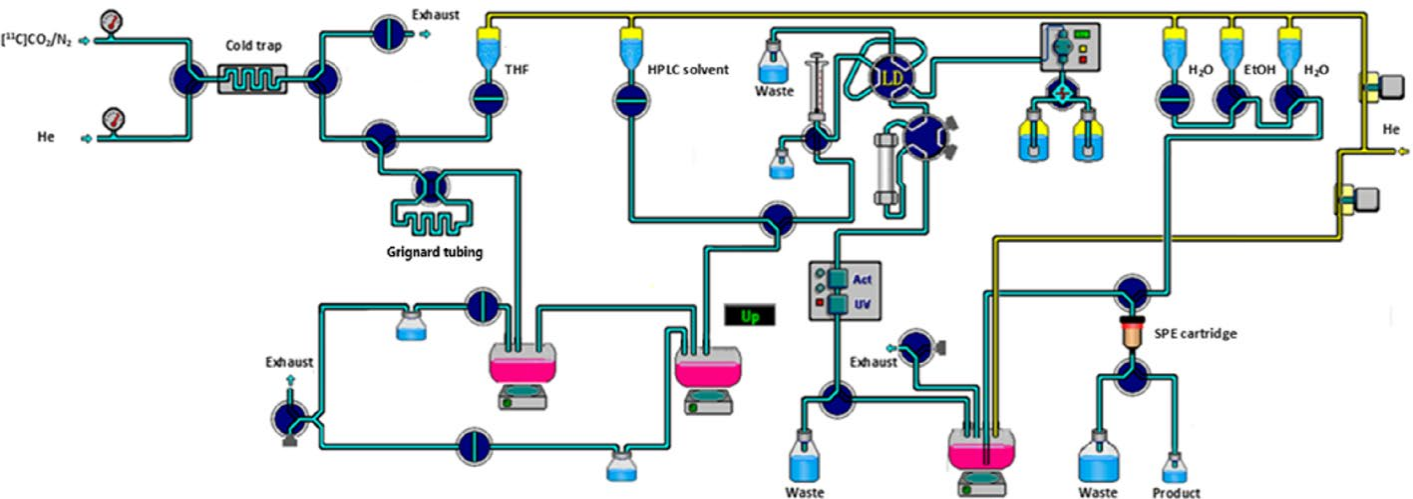
Schematic representation of the fully automated system used for [^11^C]fentanyl production

Ethylmagnesium bromide solution in diethyl ether (3 M, 500 μL) was diluted with freshly distillated THF (500 μL) in the glove box 10 min prior to [^11^C]CO_2_ delivery. The resulting solution was flushed through the polyethylene tube (0.034 inch I.D., 0.060 inch O.D., Scientific commodities, Inc.). After excess volume of solution was removed by flushing with nitrogen gas, the tube related to a 4-port 2-way valve (V-101D, IDEX Health & Science) in a closed position for the loop. This valve was installed as shown in Fig. 1. C-11 labeled carbon dioxide ([^11^C]CO_2_) was produced from the on-site cyclotron (GE PET trace 800, GE Healthcare, OH) by the ^14^N (p,α)^11^C nuclear reaction using the nitrogen target containing trace of oxygen (1%) and cryogenically trapped in a stainless coil (Length, 200 mm; OD, 1/16″; ID, 0.7 mm) at -185 °C. After radioactivity within the cold trap plateaued, the cold trap temperature was increased to 100 °C and [^11^C]CO_2_ was released into the tubing using helium flow (3.5 mL/min). The carboxylation occurred for 1 min and then the contents of the tubing was eluted using THF (450 µL) into the first reaction vessel contains phthaloyl dichloride (52 µL), 2,6-di-tert-butylpyridine (62 µL), dimethylformamide (2.64 µL). The crude mixture was distilled to remove excess THF under a stream of helium (8.5 mL/min) by heating to 89 °C. After injection of chloroform (200 µL), the second distillate portion (90–130 °C) was collected to the second reaction vessel containing 4-ANPP (1 mg, 3.6 µmol), chloroform (50 µL) and *N*,*N*-diisopropylethylamine (DIPEA, 8 µL, 46 µmol). The second reaction vessel was heated to 60 °C and maintained for 5 min for [^11^C]fentanyl synthesis. Afterwards, chloroform was removed by heating to 100 °C under helium flow (8.5 mL/min), followed by cooling down to 30 °C. The crude mixture was diluted (HPLC solvent (900 µL) mixed with 12 M HCl (5 µL)) and purified by semi-preparative HPLC (column, Chromolith RP-18 monolithic HPLC column, 100 × 10 mm, 5 μm; mobile phase: 0.01 M hydrochloric acid/ethanol = 80/20; flow rate, 5 mL/min; detection wavelength, 210 nm) (Fig. S1). [^11^C]Fentanyl was collected at 11 min and pH was adjusted with 1 M NaOH solution and sodium phosphate buffer.

### Small animal PET studies

Anesthesia was initially induced using isoflurane (5%) in a stream of oxygen gas (1.25 L/min) for 5 min, then maintained at low isoflurane (1–2%) throughout the study. A catheter connected with a tubing (BTPE-10, 48 cm; Instech Laboratories, Inc., PA) was inserted into the tail vein. After a CT scan, a dynamic PET scan was performed in a list mode for 90 min simultaneously from the start of [^11^C]fentanyl administration in one-minute bolus (488 ± 567 µCi, 284 ± 140 µL) using a PHD 2000 syringe pump (Harvard Apparatus, Holliston, MA). Vital signs were measured with a Physiosuite or MouseStat (Kent Scientific., Torrington, CT). The acquired dynamic PET data was reconstructed into time 23 frames (6 × 20 s, 5 × 60 s, 4 × 120 s, 3 × 300 s, 3 × 600 s, 2 × 900 s). Elacridar (3 mg/kg; TargetMol Chemicals Inc., Boston, MA) was prepared and administrated at 15 min prior to [^11^C]fentanyl injection as shown in the previous literature (Tang et al. 2024).

### Ex vivo biodistribution studies: radiometric HPLC analysis

[^11^C]Fentanyl was intravenously injected into anesthetized Wistar rats (2764 ± 1393 µCi, 650 ± 212 µL, n = 12). Blood samples (0.2 to 0.5 mL) were collected from the femoral artery through BTPU-27 tubing (Instech Laboratories, Inc., Plymouth Meeting, PA) at 1, 1.5, 3, 5, 10, 15, 30, 45, and 60 min after radiotracer injection (n = 1–6 per time point). Samples were processed for both radiolabeled metabolite analysis and total radioactivity quantification. For metabolite analysis, each blood sample was centrifuged at 14,500 RPM for 2 min (Eppendorf MiniSpin Centrifuge, Enfield, CT). The resulting plasma samples were mixed with equal volume of acetonitrile and vortexed and centrifuged again to remove plasma proteins prior to radiometric HPLC analysis. In parallel, an aliquot of each plasma sample was weighed and measured using the 2480 Wizard gamma counter (Perkin Elmer, Waltham, MA) to determine total radioactivity for SUV calculations.

Radiometabolite analysis and total radioactivity quantification were also done in the brain and interscapular brown adipose tissue (BAT). Rats were sacrificed at 15, 30, 45, and 60 min after tracer administration (n = 2–4 per time point) and the brain and BAT samples were dissected, weighed, and counted with the gamma counter. Samples were then treated with acetonitrile (500 µL) and homogenized at 3000 RPM for 4 min with a homogenizer (099C K54, Glas-Col LLC, Terra Haute, IN). The mixture was centrifuged at 14,500 RPM for 2 min and supernatants were filtered through polypropylene syringe filters (Tisch Scientific, Cleves, OH) for radiometabolite analysis.

Radiometabolite analysis of plasma, brain, and BAT samples were performed with a radiometric HPLC (column, Chromolith Semi-Prep RP-18e endcapped column, 100 × 10 mm, 2 µm; mobile phase, isocratic 0.01 M HCl/EtOH = 77/23; flow rate, 5 mL/min; detection wavelength, 210 nm) equipped with a G1367C autosampler (Agilent, Wilmington, DE), two Azura P 4.1S pumps (Knauer, Berlin, Germany), a BlueShadow detector 10D at 210 nm (Knauer, Berlin, Germany), and a radiodetector (B-FC-4100 BGO High Voltage Detector) paired with a Colibrick AD converter (DataApex, Prague, Czechia) (Fig. S2).

### Pharmacokinetic analysis in plasma

Pharmacokinetic parameters were generated using a 2-compartment model in a Microsoft Excel Add-in, PKSolver (PMID: 20,176,408). A weighting of 1/C_p_^2^ was utilized for fitting to the biexponential function, where C_p_ was the plasma concentration.

### PET image processing and statistical analysis

Reconstructed PET data was co-registered to the rat brain atlas (Schiffer et al. 2006) in PMOD (v2.8 PMOD Technologies, Zurich, Switzerland) and the resulting parameters applied into the corresponding dynamic PET data. Time-activity curves were generated using a regions of interest (ROIs) template and expressed as standard uptake values (SUV). Volumes of interest (VOIs) of peripheral organs were manually identified based on CT and PET images. Results are reported as mean ± standard deviation and analyzed in Microsoft Excel. To compare brain regions within rats a repeated measures one-way ANOVA was performed. Unpaired t-tests were performed for comparison between controls mice and the efflux transporter knockout mice and elacridar pretreatment animals.

## Results

### Radiosynthesis of [^11^C]Fentanyl

All the steps for [^11^C]fentanyl radiosynthesis were applied to the commercially available radiochemistry module equipped with minor modifications as shown in Fig. 1. The averaged total synthesis time was about 42 min (n = 5), providing moderate radiochemical yield (10.4 ± 5.7%, decay corrected, n = 5) and high radiochemical purity (> 99%). Sufficient [^11^C]fentanyl (13.2 ± 7.0 GBq, n = 5) at the end of synthesis was routinely produced from ~ 129.5 GBq (~ 3.5 Ci) of [^11^C]CO_2_. Molar activity ranged from 384.8 to 2571.5 GBq/μmol (10.4 to 69.5 Ci/μmol) at the end of bombardment. Co-injection of the nonradioactive fentanyl with [^11^C]fentanyl in analytical HPLC system showed identity of the product was well established (retention time, 8.9 min) (Fig. S3).

### PET study: brain pharmacokinetics and influence of brain efflux pumps

**Fig. 2:**
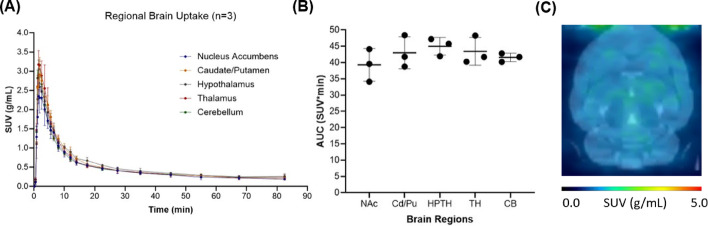
Baseline pharmacokinetics of [^11^C]fentanyl in the brain. **A** Averaged time-activity curves of [^11^C]fentanyl in standard uptake value (SUV, g/mL) and **B** The area under the time activity curve (AUC) for the nucleus accumbens (Nac), caudate/putamen (Cd/Pu), hypothalamus (HPTH), thalamus (TH), and cerebellum (CB). **C** A group-averaged standard uptake value (SUV) image (averaged from 0 to 15 min)

Whole brain pharmacokinetics of [^11^C]fentanyl in the rat brain was characterized by fast and high uptake (SUV_max_ = 2.71 ± 0.122 g/mL, T_max_ = 1.72 min) and fast clearance (T_1/2_ = 5.06 min) (Fig. 2A). Brain uptake was largely homogenous across cortical and subcortical brain regions; consistently, the area under the time-activity curves (Fig. 2B) did not differ significantly in five brain regions (one-way ANOVA, p = 0.1383) as also shown in the averaged PET image from 0 to 15 min (Fig. 2C).

**Fig. 3:**
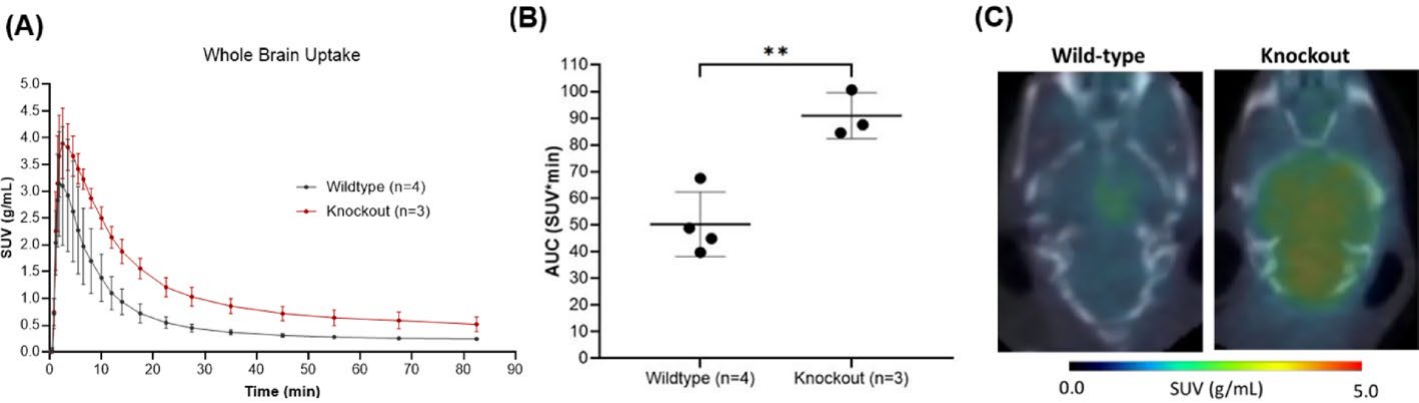
Whole brain uptake of [^11^C]fentanyl in P-gp and BCRP KO mice versus wildtype mice. **A** Averaged time-activity curve of [^11^C]fentanyl in the brain of KO mice and wildtype mice. **B** Area under the time-activity curve for brain uptake of [^11^C]fentanyl in KO mice and wildtype mice. **C** Representative brain uptake difference between KO and wildtype mice

The effect of efflux transporters on [^11^C]fentanyl brain pharmacokinetics were measured in P-gp and BCRP KO mice and compared to wildtype mice. KO mice had higher whole-brain peak uptake (n = 3, SUV_max_ = 3.94 ± 0.629 g/mL), later peak brain uptake (T_max_ = 2.50 min) and slower clearance (T_1/2_ = 14.1 min) in comparison to wildtype mice (n = 4, SUV_max_ = 3.17 ± 1.04 g/mL, T_max_ = 1.83 min, T_1/2_ = 8.96 min) (Fig. 3A). This resulted in higher [^11^C]fentanyl exposure in the KO’s brain, observed by a higher area under the time-activity curve relative to wildtype mice (Fig. 3B). This area under the curve difference was significant (unpaired t-test, p = 0.0044) and can be visualized in the averaged SUV image (0 to 15 min) from a KO and a wildtype mouse (Fig. 3C).

**Fig. 4:**
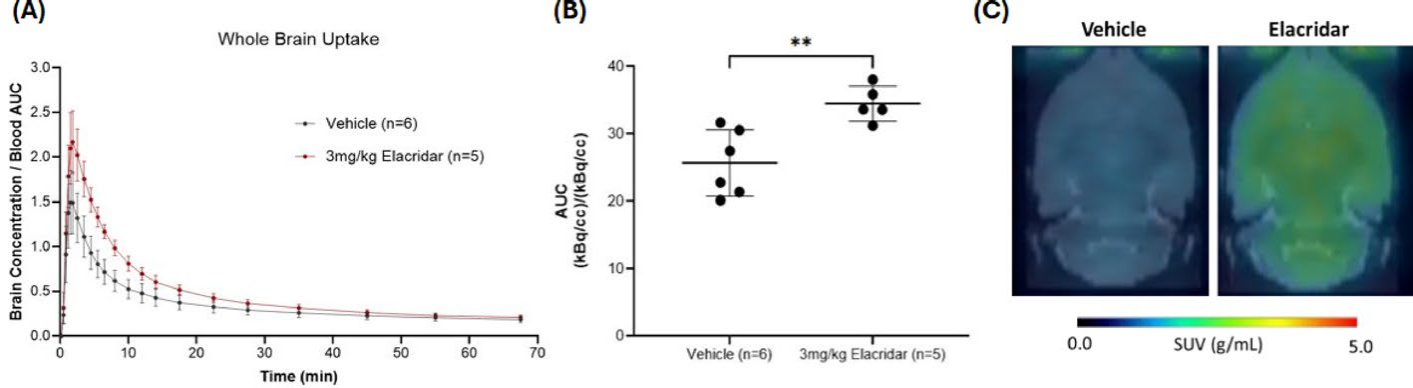
Whole brain uptake of [^11^C]fentanyl in Wistar rats pretreated with 3 mg/kg elacridar or vehicle. **A** Averaged time-activity curve of [^11^C]fentanyl in whole brain of elacridar and vehicle pretreated rats. **B** Area under the time-activity curve for brain uptake of [^11^C]fentanyl in elacridar and vehicle pretreated rats

For rats, when P-gp and BCRP efflux transporters were blocked by pretreatment with a 3 mg/kg dose of elacridar, the brain uptake of [^11^C]fentanyl was higher and peaked later (n = 5, brain/blood max = 2.18 ± 0.367, T_max_ = 1.83 min) and its clearance was slower (T_1/2_ = 7.27 min) than the animals pretreated with vehicle (Fig. 4A). The elacridar pretreated group showed a significantly higher AUC value in comparison to vehicle (unpaired t-test, *p* = 0.0059) (Fig. 4B), also seen in the averaged SUV image (0 to 15 min) (Fig. 4C).

### PET study: whole-body pharmacokinetics and ex vivo radiometric HPLC analysis

**Fig. 5:**
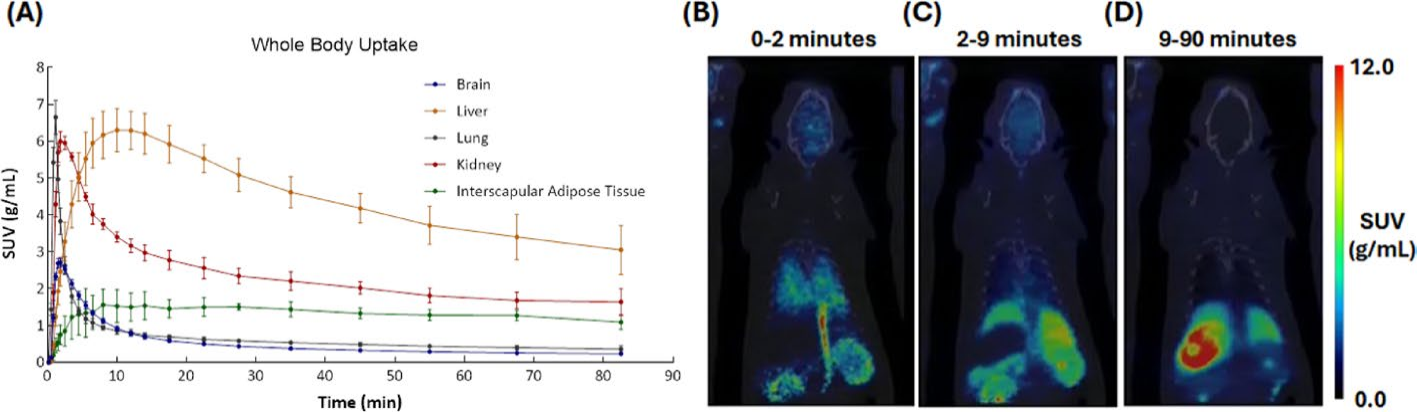
Whole-body pharmacokinetics of [^11^C]fentanyl in Wistar rats. **A** Averaged time-activity curves of [^11^C]fentanyl in the brain, liver, lung, kidney and interscapular adipose tissue (n = 3). Standard uptake value (SUV) images of a representative rat time-averaged at **B** 0–2 min, **C** 2–9 min, and **D** 9–90 min

Whole body imaging in rats showed rapid [^11^C]fentanyl uptake in lungs (T_max_ = 1.17 min) and kidneys (T_max_ = 1.83 min) (Fig. 5A and B) while C-11 uptake in the liver was slower (T_max_ = 10 min) (Fig. 5A and C), but was the highest of all organs. Clearance of [^11^C]fentanyl was slow in the kidney (T_1/2_ = 11.2 min) and slower in liver (T_1/2_ = 59.1 min) (Fig. 5A and D). The interscapular adipose tissue showed slow peak uptake (SUV_max_ = 1.73 ± 0.313 g/mL, T_max_ = 8 min) and had the slowest clearance of all organs/tissues (T_1/2_ = 177 min) (Fig. 5A).

**Fig. 6:**
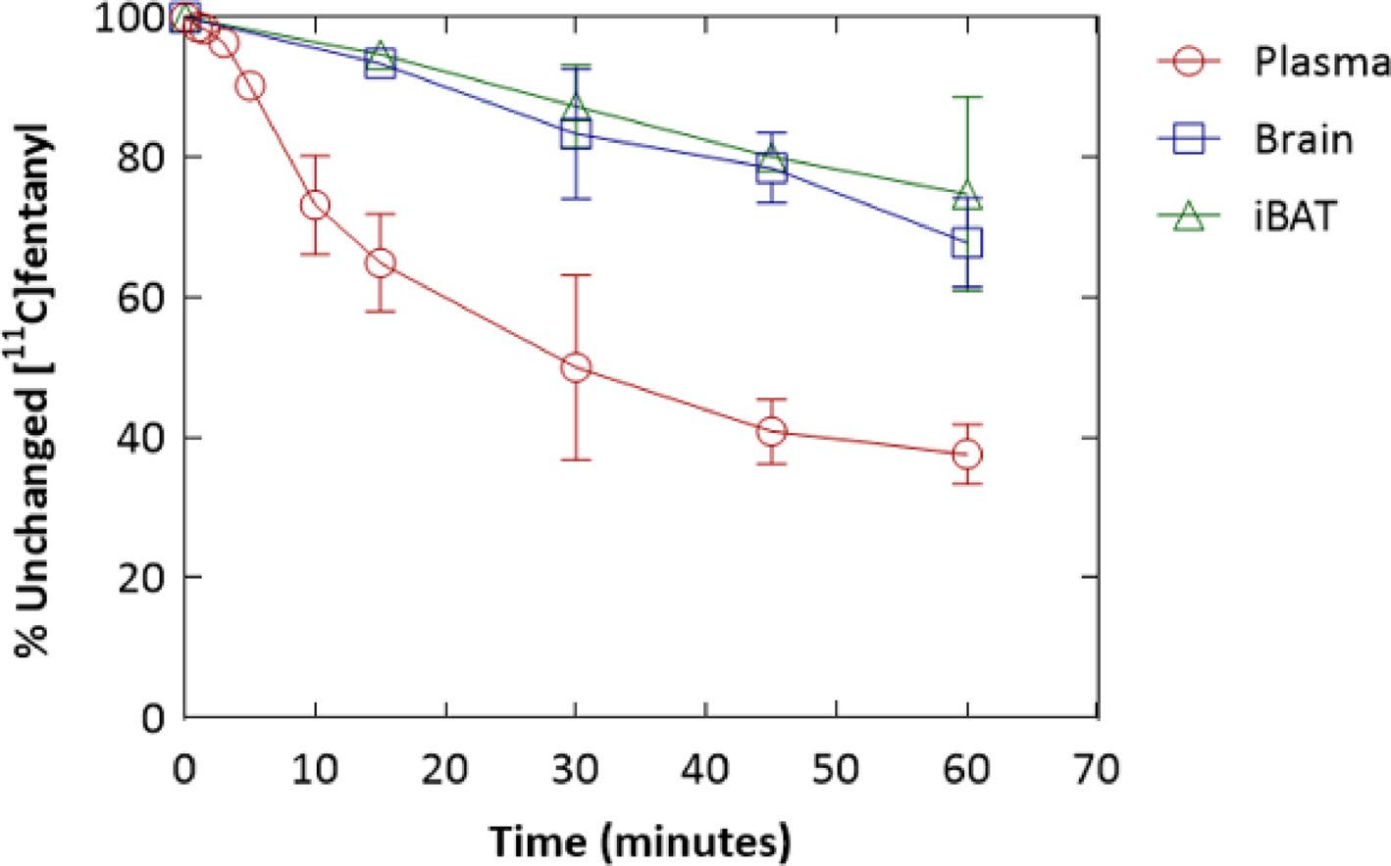
Metabolism of [^11^C]fentanyl in the plasma, brain, and interscapular brown adipose tissue (iBAT) of Wistar rats represented as percentage of unchanged [^11^C]fentanyl over time

**Fig. 7:**
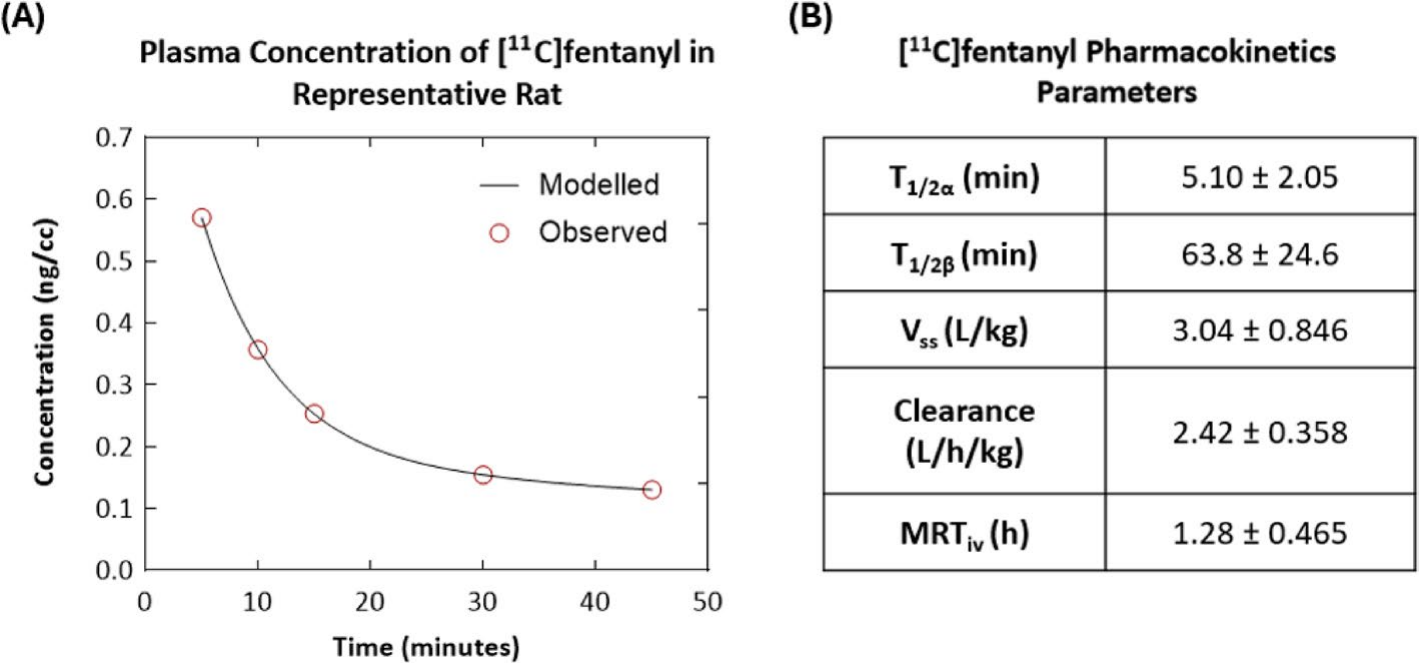
Plasma pharmacokinetics of [^11^C]fentanyl in Wistar rats. **A** Metabolism corrected plasma concentration (ng/cc) of [^11^C]fentanyl in a representative rat over time (C_p_(t) = Ae^−αt^ + Be^−βt^ = 0.824e^−0.147t^ + 0.181e^−0.008t^). **B** [^11^C]fentanyl pharmacokinetics parameters calculated from plasma (n = 3)

Ex vivo analysis demonstrated that, 30 min after [^11^C]fentanyl injection, less than 50% of total activity in the plasma remained as parent radioactivity. In contrast, a significantly greater proportion of radioactivity remained as unmetabolized parent tracer in the brain (83%) and brown adipose tissue (BAT) (87%) (Fig. 6). The plasma pharmacokinetics of [^11^C]fentanyl plotted as time versus plasma concentration (ng/cc) showed a bi-phasic decline (Fig. 7A). Parameter estimation of [^11^C]fentanyl’s pharmacokinetics in plasma revealed short half-lives for the distribution (5.10 ± 2.05 min) and elimination (63.8 ± 24.6 min) phases and a high volume of distribution (3.04 ± 0.846 L/kg) at steady state (Fig. 7B).

## Discussion

### Radiosynthesis

**Scheme 1:**
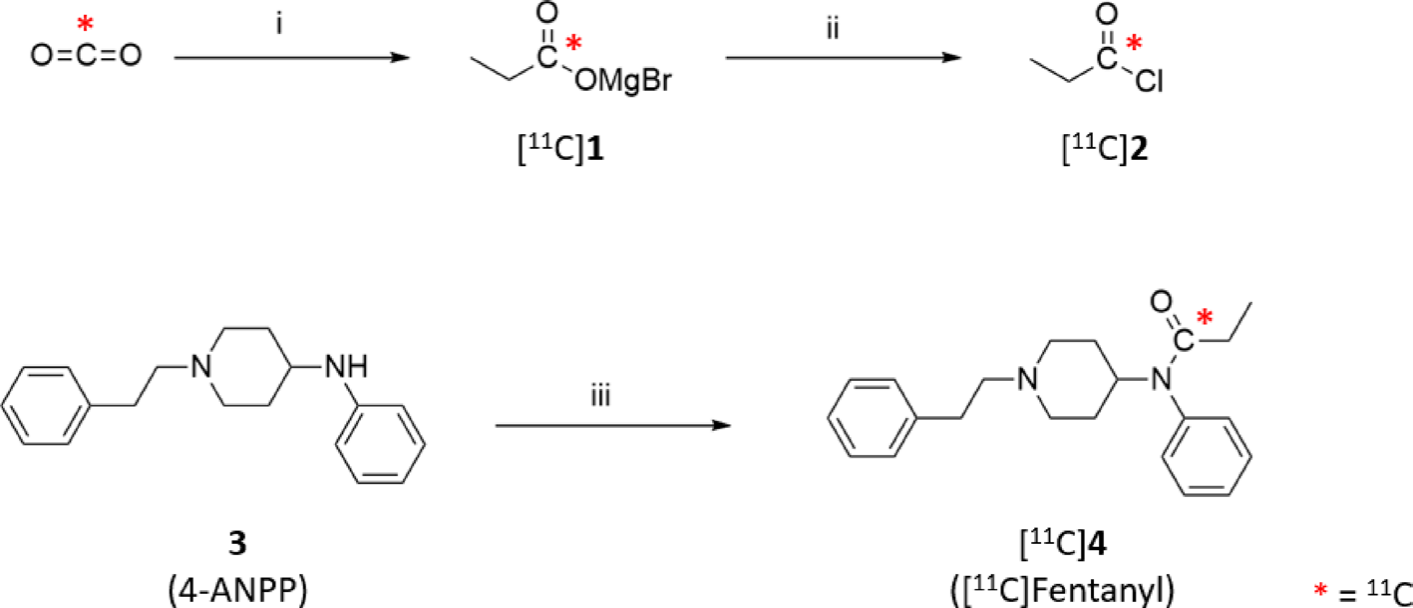
Radiosynthesis of [^11^C]fentanyl ([^11^C]**4**): (i) ethylmagnesium bromide in ether/THF impregnated in polyethylene tubing, R.T., 1 min, (ii) phthaloyl dichloride, 2,6-di-*tert*-butylpyridine, DMF, THF, 130 °C, (iii) [^11^C]propionyl chloride ([^11^C]**2**), DIPEA, chloroform, 60 °C, 5 min

In this study, the radiolabeling of fentanyl with carbon-11 was achieved through a three-step, two-pot process: (1) [^11^C]carboxylation of ethylmagnesium bromide, (2) generation and distillation of [^11^C]propionyl chloride, and (3) [^11^C]propionylation of 4-ANPP (Scheme 1). Initial attempts to use a previously reported one-pot “in-loop” carboxylation and amidation protocol with triethylamine (TEA) in THF resulted in low and inconsistent radiochemical yields, and a requirement for excess precursor (> 7 µmol), which complicated the purification process (Rami-Mark et al. 2013). These results are likely due to the use of excess thionyl chloride and the low nucleophilicity of the anilinic amine group of 4-ANPP.

Since Pike et al. (Luthra et al. 1985) introduced the two-pot [^11^C]acylation approach, a key radioactive precursor, [^11^C]acyl chloride has been utilized in the synthesis of various radiotracers including [^11^C]diprenorphine (Luthra et al. 1985), [^11^C]buprenorphine (Luthra et al. 1987), [^11^C]ohmefentanyl (Zhu et al. 1992), [^11^C]pyrazosin (Ehrin et al. 1988), [^11^C]-( +)-PHNO (Pfaff et al. 2019), [^11^C]WAY-100635 (McCarron et al. 1996), [^11^C]cyclophan (McPherson et al. 1986), [^11^C]melatonin derivatives (Bars et al. 1987), and [^11^C]physostigmine (Bonnot-Lours et al. 1992). We selected the in-loop carboxylation and distillation of [^11^C]acyl chloride to improve molar activity and reduce interference from excess chlorinating reagent, thereby minimizing the amount of amine precursor required for amidation. Additionally, this two-pot strategy allowed for the optimization and monitoring of each step via radiometric analysis.

For [^11^C]carboxylation, a loop containing the Grignard reagent (37 μL) was prepared in a glove box using a 4-port 2-way valve, and installed to the radiochemistry module. This procedure strictly excluded ambient carbon dioxide to minimize C-12 mass introduction, which likely contributed to achieving the exceptionally high molar activity (up to 70 Ci/μmol). While trapping efficiency of C-11 radioactivity was > 99% in the loop, the concentration of the Grignard reagent (GR) was critical for production of [^11^C]propionate. As reported in prior studies (Rami-Mark et al. 2013; Zhu et al. 1992; McCarron et al. 1996), the concentration of the GR is critical; low concentrations led to poor [^11^C]CO_2_ conversion, while high concentrations resulted in overreaction. Radiometric HPLC analysis confirmed the formation of [^11^C]diethyl ketone as a byproduct and the presence of unreacted [^11^C]CO_2_ (Fig. S4). 1.5 M of GR concentration showed 46% of [^11^C]propionic acid and 10% of [^11^C]diethyl ketone in total 56% of [^11^C]CO_2_ conversion.

The crude [^11^C]propionyl chloride, generated from [^11^C]propionate using phthaloyl dichloride, was distilled by heating under a stream of helium. The radioactivity in the initial distillate fraction (20–90 °C) accounted for only 1.4–10.5% (n = 5) of the total. Thus, the second fraction (90–130 °C) was used for subsequent amidation, dramatically reducing the solvent volume in the second reaction vessel. The distilled [^11^C]propionyl chloride represented 21 ± 8% (n = 5) of the total radioactivity in the first reaction vessel. The remaining activity (27.6 ± 8%, n = 5) could not be distilled, even at temperatures up to 180 °C.

As previously mentioned, the low radiochemical yield observed during the acylation of 4-ANPP is likely due to the poor nucleophilicity of its anilinic amine (Cai et al. 2012). In contrast, more nucleophilic amines such as 1-(4-methoxyphenyl)piperazine exhibited high [^11^C]propionylation yield (> 30%, data not shown). To improve yields with 4-ANPP, various solvent and base combinations were systematically screened using non-radioactive ("cold") propionyl chloride and 4-ANPP under short reaction times (Fig. S5, S6). Among the organic bases tested, DIPEA and 1,2,2,6,6-pentamethylpiperidine showed highest yields in both chloroform and THF. Solvent screening with DIPEA revealed that polar chlorinated solvents such as chloroform and dichloromethane were most effective. Based on these results, [^11^C]propionylation conditions were directly compared to TEA/THF condition (Table 1). While DIPEA/THF gave moderate yield (56.9 ± 10.2%, n = 5), DIPEA/chloroform gave slightly higher yield (62.5 ± 11.3%, n = 3). However, TEA provided very poor yield (7.7%) regardless of solvents, which is consistent with the nonradioactive version of test results.

**Table 1:** [^11^C]Fentanyl synthesis parameters

Amidation condition	CO2 trapping efficiency (%)*	Elution efficacy (%)**	Distillation efficacy (%)***	[^11^C]Amidation yield (%)****	Radio chemical yield (%)*****
THF, DIPEA (n = 5)	92.5 ± 2.6	99.2 ± 1.2	43.1 ± 11.0	56.9 ± 10.2	10.4 ± 5.7
Chloroform, DIPEA (n = 3)	96.1 ± 2.4	92.0 ± 7.5	29.0 ± 6.0	62.5 ± 11.3	34.3 ± 12.9
Chloroform, TEA (n = 1)	96.6	95.0	6.8	7.7	3.3

^*^Each part of activity in the system was measured to estimate total [^11^C]CO_2_ and [^11^C]CO_2_ trapped in Ascarite II trap, **Percentage of activity eluted from Grignard reagent immobilized polyethylene tube, ***Percentage of activity that distilled from first reaction vessel to second reaction vessel, ****Based on crude sample injection results from analytical HPLC, *****Based on the total activity of [^11^C]CO_2_ at EOB

### Preclinical PET studies

Understanding fentanyl’s pharmacokinetics throughout the various organs/tissues is invaluable, as its clinical effects, toxicity, and duration of action are determined by its concentrations at specific target sites rather than by plasma levels alone. Organ/tissue-specific data may reveal how fentanyl’s rapid distribution to the brain underlies its fast-acting analgesic and respiratory depressant effects as well as its almost immediate rewarding effects, while its subsequent redistribution to peripheral organs can influence residence time and the pattern of elimination. Knowledge of tissue-level pharmacokinetics thus informs the clinical management of fentanyl toxicity and enhances our understanding of its biodistribution, especially with chronic or high-dose use, ultimately supporting improved therapeutic strategies such as opioid overdose reversal interventions.

Our PET imaging results demonstrate rapid and high brain penetration of [^11^C]fentanyl, consistent with its fast onset of analgesia and the risk of acute respiratory depression when misused; the estimated brain AUC was approximately three times of plasma AUC. Fentanyl distribution in the brain was widespread and did not preferentially accumulate in opioid receptor-rich regions, suggesting largely non-specific signals. This was further supported by naloxone pretreatment studies, which showed no significant change in brain uptake or regional distribution (data not shown). Both brain permeability and clearance were significantly altered by inhibition or genetic knockout of two major efflux pumps, resulting in increases of up to 34% in rats and 81% in mice. These findings are consistent with previous reports indicating that fentanyl is a substrate for P-gp and BCRP, and that efflux pump inhibition is associated with enhanced central effects and respiratory depression (Yu et al. 2012). This is particularly relevant given that chronic exposure to opioid drugs alters the expression efflux transporters of cerebral blood vessels (Schaefer et al. 2018).

Peripherally, [^11^C]fentanyl PET showed initial high uptake in the lung, heart, liver, and kidneys. Notably, uptake in brown adipose tissue (BAT) increased gradually and remained elevated throughout the 90-min scan, with BAT concentrations exceeding those in the brain. The prolonged retention and higher concentration of fentanyl in adipose tissues suggest that fat may serve as a reservoir, delaying fentanyl clearance from the brain and potentially contributing to re-narcotization following opioid reversal. This mechanism may be particularly important in chronic fentanyl users, where delayed elimination could complicate overdose management. Indeed, the re-narcotization observed after fentanyl overdose reversal with naloxone in some fentanyl misusers is believed to reflect fat accumulation from repeated exposures consistent with the presence of fentanyl in urine for up to 1 week in fentanyl misusers (Bird et al. 2023).

Experimental data on fentanyl concentrations in human brain and peripheral tissues are extremely limited, primarily derived from postmortem forensic studies and a small number of intraoperative CSF measurements. The use of [^11^C]fentanyl for PET imaging presents a valuable tool to noninvasively quantify fentanyl pharmacokinetics in various human populations and clinical scenarios, providing better understanding of fentanyl disposition and its clinical implications.

## Conclusion

[^11^C]Fentanyl was reliably synthesized in high molar activity, effectively minimizing isotopic dilution through an automated two-pot synthesis. Rodent PET imaging demonstrated rapid and high brain penetration, with evidence of interaction with brain efflux transporters in vivo. Furthermore, our findings revealed prolonged accumulation of [^11^C]fentanyl in adipose tissues, suggesting a significant peripheral reservoir. These data indicate that [^11^C]fentanyl could serve as a valuable tool for understanding fentanyl’s brain and whole-body pharmacokinetics across diverse patient populations. [^11^C]Fentanyl may be crucial for developing personalized therapeutic strategies, as drug disposition can be significantly influenced by individual factors including age, sex, genetics, and organ function. This approach is particularly relevant for patients with metabolic abnormalities, opioid use disorder, or obesity, where fentanyl uptake, distribution, and clearance may differ substantially. Additionally, it holds promise for special populations such as children, who have distinct metabolic and blood–brain barrier characteristics, and pregnant patients, in whom fentanyl can cross the placenta and affect the fetus. By enabling direct, noninvasive quantification of fentanyl concentrations in key tissues, [^11^C]fentanyl PET can help optimize dosing, improve safety, and support more effective, individualized opioid therapy.

## Supplementary Information

Below is the link to the electronic supplementary material.

Supplementary Material 1

## Data Availability

The datasets generated and/or analyzed during the current study are available from the corresponding author upon reasonable request.
