# Supplementary material for: [11C]Fentanyl: radiosynthesis and preclinical pet imaging for its pharmacokinetics
Source: EJNMMI Radiopharm Chem. 2025 Oct 28;10:70. doi: 10.1186/s41181-025-00394-z (PMC12569255; doi:10.1186/s41181-025-00394-z)
Supplement: Supplementary file 1 — Supplementary Material 1 [file 41181_2025_394_MOESM1_ESM.docx]

**[^11^C]Fentanyl: Radiosynthesis and Preclinical PET Imaging for Its Pharmacokinetics**

European Journal of Nuclear Medicine and Molecular Imaging Radiopharmacy and Chemistry

Woochan Kim^1^, Aaron K. Wozniak^1,^*, Nathaniel J. Burkard^1,^*, Michael L. Freaney^1^, Ailen Costamagna-Soto^1^, Kelly O’Conor^1^, Abolgasem Bakhoda^1^, Seth M. Eisenberg^1^, Wenjing Zhao^1^, Jeih-San Liow^2^, Nora D. Volkow^1,&^, Sung Won Kim^1,&^

*Both authors contributed equally

^&^Corresponding authors

^1^Laboratory of Neuroimaging, National Institute on Alcohol Abuse and Alcoholism, National Institutes of Health, Bethesda, MD 20892, USA. ^2^Molecular Imaging Branch, National Institute of Mental Health, National Institutes of Health, Bethesda.

**E-mail addresses**:

[kimw9@nih.gov](mailto:kimw9@nih.gov), [aaron.wozniak@nih.gov](mailto:aaron.wozniak@nih.gov), [nate.burkard@nih.gov](mailto:nate.burkard@nih.gov), [michael.freaney@nih.gov](mailto:michael.freaney@nih.gov), [ailen.costamagna-soto@nih.gov](mailto:ailen.costamagna-soto@nih.gov), [kellyoconor@hms.harvard.edu](mailto:kellyoconor@hms.harvard.edu), [abakhoda@towson.edu](mailto:abakhoda@towson.edu), [sethmeisenberg@gmail.com](mailto:sethmeisenberg@gmail.com), [zhaowellen@gmail.com](mailto:zhaowellen@gmail.com), [liowj@mail.nih.gov](mailto:liowj@mail.nih.gov), nvolkow@nida.nih.gov, sunny.kim@nih.gov

***Correspondence to**:

Nora Volkow, M.D. and Sung Won Kim, Ph.D.

National Institute on Alcohol Abuse and Alcoholism (NIAAA) National Institutes of Health

10 Center Drive, Room B2L124: MSC 1013

Bethesda , MD 20892-1013

+1 (301) 402-0868

**Semi-preparative HPLC chromatogram**


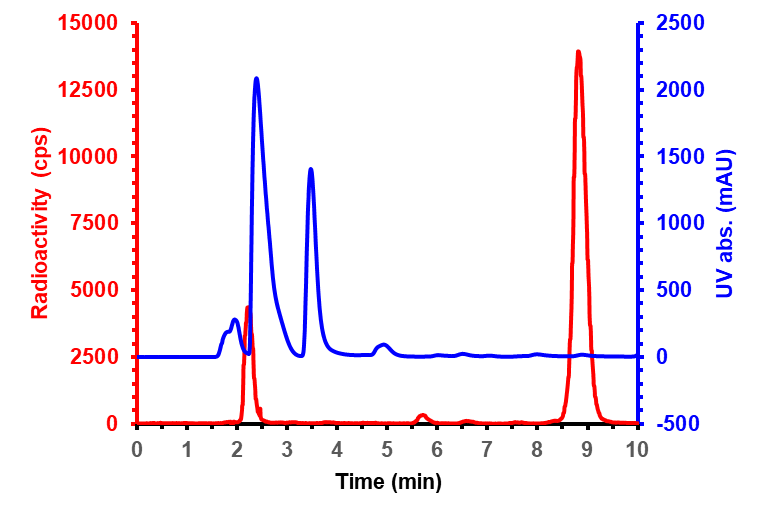


[^11^C]fentanyl

**Fig. S1** Representative semi-preparative HPLC profile of [^11^C]fentanyl. Analysis performed on using a Knauer HPLC pump (5 ml/min), equipped with a Chromolith RP-18 monolithic HPLC column, 100x10 mm, 5 μm; mobile phase: 0.01 M hydrochloric acid/ethanol = 80/20; flow rate, 5 mL/min; detection wavelength, 210 nm).

**[^11^C]Fentanyl metabolite study**


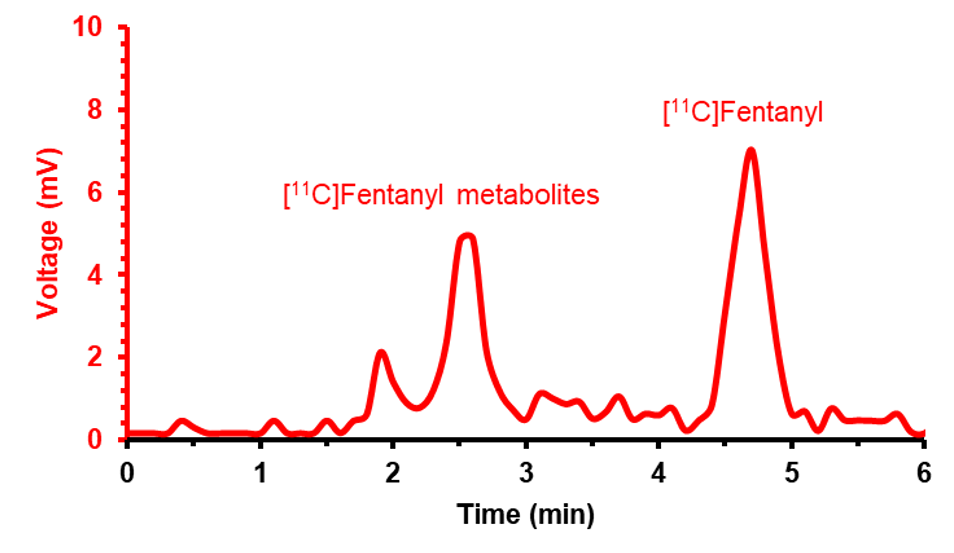


**Fig. S2** Representative radiochromatogram of [¹¹C]fentanyl and its metabolites in rat plasma. Radiometabolite analysis was performed using a radiometric HPLC system (Agilent 1100 Series) equipped with a Chromolith Semi-Prep RP-18e endcapped column (100×10 mm, 2 µm). An isocratic mobile phase consisting of 0.01 M HCl/ethanol (77:23, v/v) was used at a flow rate of 5 mL/min.

**Analytical HPLC chromatogram**


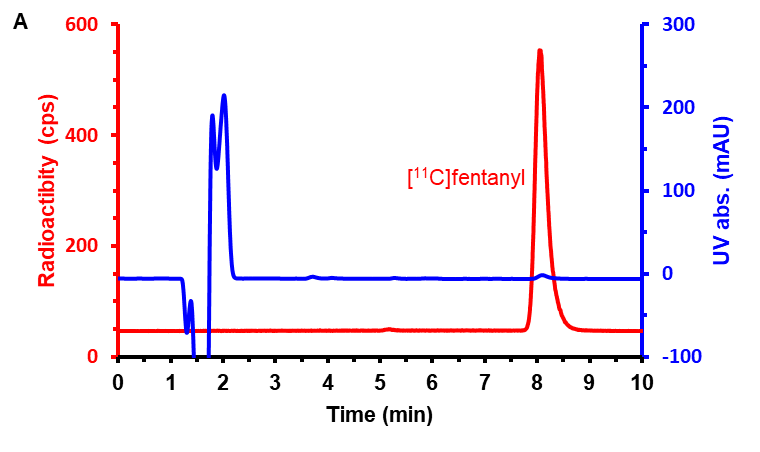


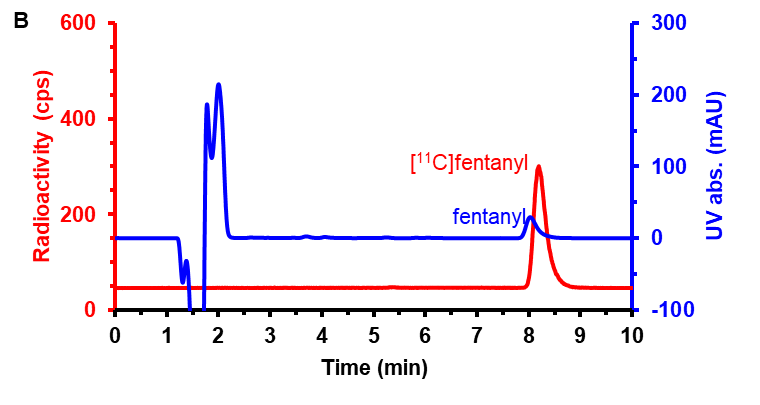


**Fig. S3** A. Analytical HPLC UV and radiochromatogram of [^11^C]fentanyl at 210 nm. B. Analytical HPLC co-injection chromatogram of [^11^C]fentanyl with 0.1 ug of fentanyl. Analysis performed on an Agilent 1100 series HPLC equipped with a Agilent Eclipse XDB C-18 column (150x4.6 mm, 5 µm) and 0.1% TFA aqueous solution/ACN = 70/30 in one pot (pH 2.0) was used as a mobile phase with a flow rate of 1 mL/min.

**[^11^C]CO_2_ conversion result with 1.5 M ethylmagnesium bromide in THF/diethyl ether**


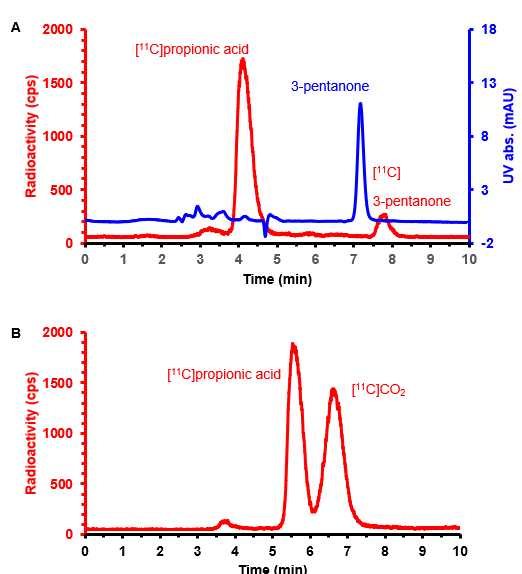


**Fig. S4** A. Analytical HPLC co-injection UV (270 nm) and radiochromatogram of [^11^C]synthon with 3-pentanone (1 μg) as the result of polyethylene tube elution with THF (450 μL) after [^11^C]carboxylation of ethylmagnesium bromide (37 μL of 1.5 M ethylmagnesium bromide THF/diethyl ether solution was impregnated in the tubing). B. Analytical HPLC injection chromatogram of the [^11^C]synthon after the [^11^C]carboxylation. Analyses were performed using an Agilent 1100 series HPLC system equipped with a Phenomenex Luna C18 column (250×4.6 mm, 10 µm). The mobile phase for panel A was 0.1% TFA in water/acetonitrile (72:28, v/v), and for panel B, 0.1% TFA in water/acetonitrile (90:10, v/v), each at a flow rate of 1 mL/min. Two different solvent systems were used to better resolve the radioactive peak profile.

**Amidation Optimization**


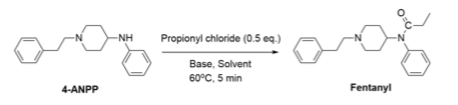
4-ANPP (3.6 µmol) was reacted with propionyl chloride (1.8 µmol, 10 times diluted in reaction solvent immediately prior to beginning the reaction) in the presence of a base (46.4 µmol). For all reactions, 4-ANPP was dissolved in the reaction solvent to make a stock solution of 1 mg/50 µL. 50 µL of 4-ANPP stock solution was then added to a reaction vessel with enough solvent to make the final reaction volume 130 µL, followed by the addition of a base. Each reaction vessel was vortexed before adding diluted propionyl chloride and immediately placed in an oil bath at 60°C for five minutes. Upon reaction completion, samples were quenched on dry ice and analyzed using HPLC (Agilent Eclipse XDB C-18 column, 150 x 4.6 mm, 5 µm with 0.1% TFA aqueous solution/ACN = 70/30) by 1 µL injection (λ = 210 nm).

**
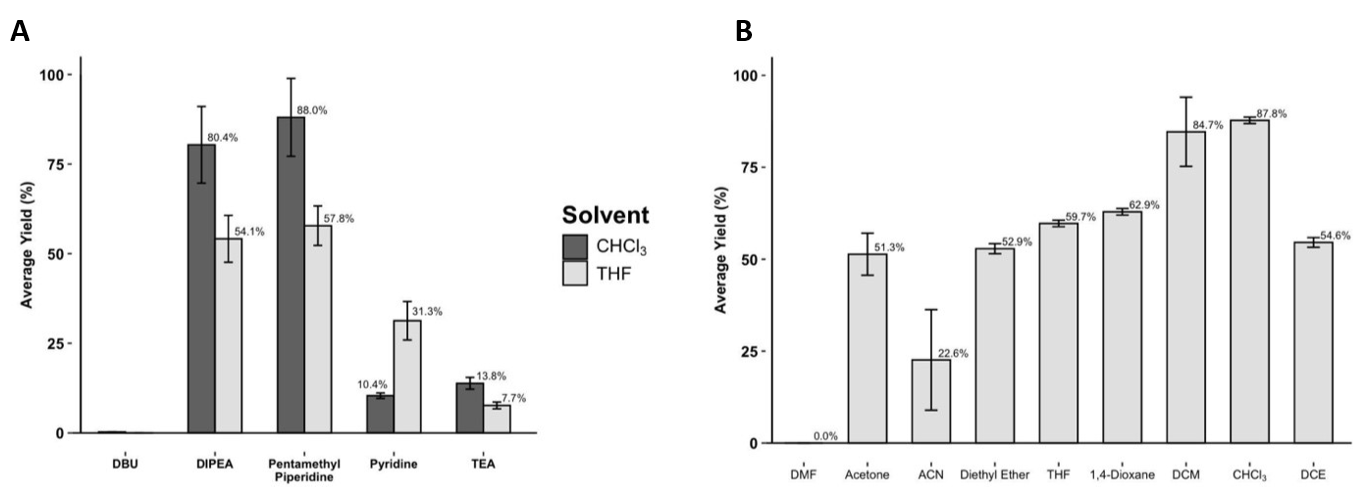
**

**Fig. S5** Average fentanyl chemical yield according to base (A) and solvent (B): 4-ANPP (3.6 µmol), propionyl chloride (1.8 µmol), base (46 µmol), temperature = 60 ºC, time = 5 minutes, n = 3. Solvent testing was conducted using DIPEA (46 µmol, 13 eq.).

***Base Selection***

Amidation reactions were performed to determine the most optimal condition for [^11^C]fentanyl amidation. Diisopropylethylamine (80.37%, 54.1%) and 1,2,2,6,6-pentamethylpiperidine (88.0%, 57.8%) show highest yields in both CHCl_3_ and THF. Previous literature showed similar reactions were conducted with a reaction time of 3.5 to 10 minutes. During initial testing no significant differences in reaction yield were observed for conditions reacting beyond 5 minutes; additionally, reactions occurring above 60°C also showed no significant improvements in yield. DIPEA was chosen for [¹¹C]fentanyl production.

***Solvent Selection***

The amidation reaction condition was further tested in a variety of solvents. All reactions were prepared using DIPEA (46 µmol) and the previous method for base selection. Chemical yields showed that halogenated polar solvents like DCM (84.7%) and CHCl_3_ (87.8%) provide the highest yields among solvents tested. Overall, the combination DIPEA and CHCl_3_ provided an average yield of 84.1 ± 4.7%, and was subsequently used as the main amidation condition in [¹¹C]fentanyl radiotracer production for animal studies.

***Precursor Influence on Amidation Kinetics***


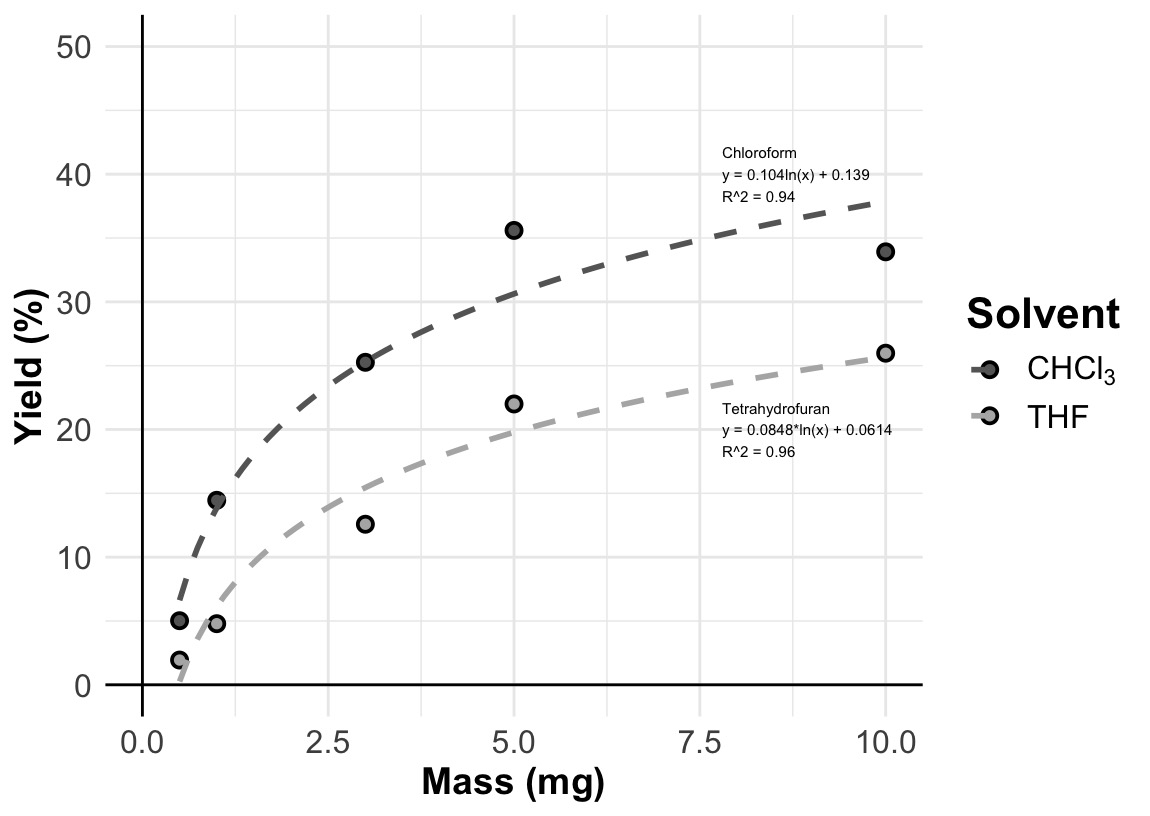


**Fig. S6** Amidation reaction kinetics: propionyl chloride (1.8 µmol), 4-ANPP (0.5, 1.0, 3.0, 5.0, 10.0), TEA (46 µmol), temperature = 60 ºC, time = 5 minutes, n = 3.

Propionyl chloride (1.8 µmol, 10 times diluted in reaction solvent immediately prior to beginning the reaction) was reacted with 4-ANPP (1.78, 3.57, 10.7, 17.8, 35.7 µmol) in the presence of a TEA (46.4 µmol). All reactions were prepared with the method described for base and solvent optimization and performed with a final reaction volume 130 µL. Upon reaction completion, samples were quenched on dry ice and analyzed using HPLC (Agilent Eclipse XDB C-18 column, 150 x 4.6 mm, 5 µm with 0.1% TFA aqueous solution/ACN = 70/30) by 1 µL injection (λ = 210 nm).

Results indicate increasing precursor quantities beyond 5 mg (10 eq.) do not produce significant changes in reaction yield. Additionally, amidation performed in chloroform shows higher yields at all equivalencies. Propionyl chloride experiments were not conducted at lower quantities due to limitations of dynamic range. The data supports that 1 mg of 4-ANPP precursor remains well above the upper limit for driving amidation reaction kinetics during [^11^C]fentanyl production, where propionyl chloride is produced at micromolar quantities.
